# Editorial: History of Neuroscience

**DOI:** 10.3389/fnana.2020.00046

**Published:** 2020-08-12

**Authors:** Jean-Gaël Barbara, Marjorie Perlman Lorch, Frank W. Stahnisch

**Affiliations:** ^1^Sorbonne Université, UPMC Université Paris 06, Institut de Biologie Paris Seine (IBPS), Neuroscience Paris Seine, UMR CNRS 8246 & Sorbonne Paris Cité, Paris Diderot, Philosophie, Histoire, SPHERE, CNRS UMR7219, Paris, France; ^2^Department of Applied Linguistics and Communication, School of Social Sciences, History and Philosophy, Birkbeck, University of London, London, United Kingdom; ^3^Alberta Medical Foundation/Hannah Professor in the History of Medicine and Health Care, University of Calgary, Calgary, AB, Canada

**Keywords:** history of neuroscience, epistemology, sociology, actor network theory, neuroscience

## History of Neuroscience—The Emergence of a Field

The History of Neuroscience is a vital field of research inquiry which brings into perspective the scholarship of the past and provides new insights into our present understanding. Since the foundational publications of *The Historical Development of Experimental Brain and Spinal Cord Physiology before Flourens* (Neuburger, 1981), *Le système nerveux central; structure et fonctions; histoire critique des théories et des doctrines* (Soury, 1899) and *The Falling Sickness: A History of Epilepsy from the Greeks to the Beginnings of Modern Neurology* (Temkin, 1945), to later contributions such as *La formation du concept de réflexe aux XVIIe et XVIIIe siècles* (Canguilhem, 1955), *The Human Brain and Spinal Cord: A Historical Study Illustrated by Writings from Antiquity to the Twentieth Century* (Clarke and O'Malley, 1967), and *A History of Neurophysiology in the Seventeenth and Eighteenth Centuries: From Concept to Experiment* (Brazier, 1984), there has been a small but growing trend in historiographical work on the neurosciences addressing a wide array of topics. These investigations have worked at the frontiers of medicine, the biological sciences, social sciences, and the humanities to explore issues in the neurological spheres of clinical care, anatomy and physiology, and behavior. The focus of this historical research spans from ancient cultures to the present day and explores biographical, institutional, disciplinary, socio-cultural, as well as technological themes and perspectives.

At the end of the twentieth century there was growing interest in the history of the neurosciences amongst clinicians and academics across many localities, institutional contexts, and disciplines. This coalesced during the 1990s with the formation of history of neuroscience research interest groups such as the ones at Louise M. Darling Biomedical Library at University of California at Los Angeles and the Wellcome Institute for the History of Medicine in London. A new generation of specialist scholars published significant monographs, including Stanley Finger's *Origins of Neuroscience: A History of Explorations into Brain Function* (Finger, 1994), Michael Hagner's *Homo cerebralis* (Hagner, 1997), or *Histoire de la neurotransmission* (Dupont, 1999). A specialist academic journal, the *Journal of the History of the Neurosciences* began publication in 1991, under the editorial leadership of Francis Schiller (1909–2003) and Frank Clifford Rose (1926–2012). Dedicated history committees of major international associations were established, including the American Academy of Neurology and the World Federation of Neurology. The trend culminated in the founding of the *International Society for the History of the Neurosciences* (ISHN) Montreal in Canada in 1995. This field of academic research continues to flourish and expand. Now history of neuroscience studies are gaining increasing academic recognition in the medical and basic sciences and are open to wider disciplinary scope beyond traditional thematic history to include sociology, anthropology and medical humanities focused on specific concepts, archives, collections and museums.

## The Individual Contributions to This Special Issue

The eight articles in this special issue span the full historical timeline of the field with the contribution by Reynolds (King's College, London) presenting novel findings on clinical Babylonian texts to those by Desmoulin-Canselier (CNRS, Nantes) and Baptiste Moutaud (Université Paris Nanterre) on the recent technique of deep brain stimulations (DBS). Many of the articles explore the nineteenth century, which was a time of great industry in the neurosciences, but much of this work is not currently well-known or appreciated. The contributions largely favor one of two opposite trends: long-term (diachronic) histories of defined subjects; or broader considerations focused on shorter periods of time. This latter trend is, for example, adopted in the biographical studies that detail disciplinary and/or sociological aspects as part of a larger context, and in the articles that take on specific disputes, paradigms and neuroscientific arguments within their time period.

All of the assembled contributions adopt both localized and geographically focused analyses placing them in general thematic perspectives. The article by Desmoulin-Canselier and Moutaud, for example, presents DBS as a case study for their enquiry on sociological and anthropological debates about medical research and basic science. Parent (Université de Laval) presents Berengario da Carpi (c. 1460–c. 1530) as a paradigmatic early Renaissance anatomist. Lorch (Birkbeck, University of London) takes the single clinical case of Henry Charlton Bastian (1837–1915) as a case to study the debates on language localization and the nuanced epistemological attitude of Bastian relative to the caricatured representations which figured in the wider European and North American debates about cortical functions which persist today. The biographical investigation of Jacques Loeb (1858–1924) by Stahnisch (University of Calgary) demonstrates the role of networks of scientists and large research centers at the turn of the twentieth century. Finkelstein (University of Colorado, Denver) presents Emil du Bois-Reymond (1818–1896) as a case of a non-naturalist physiologist concerned with the defense of Charles Darwin (1809–1882) and materialism in physiology and the life sciences in general (Finkelstein). The concept of reproductive drive developed by Franz Joseph Gall (1758–1828) is taken as an example by Paul Eling (University of Nijmegen) and Stanley Finger (Washington University in St. Louis) to demonstrate some important methodological and conceptual aspects of Gall's inquiry. Conversely, Reynolds focuses on epilepsy and demonstrates how clinical and medical thoughts, as well as neurological and psychiatric investigations, progressively built united neuroscientific perspective. Elena Giné, Carmen Martínez, and Carmen Sanz (Universidad Complutense de Madrid), Cristina Nombela (Hospital Clínico San Carlos), and Fernando de Castro (Instituto Cajal, Madrid) examine the development of the Madrid school of neurology and the role of its founder Santiago Ramón y Cajal's (1852–1934) in changing attitudes toward women in science (Giné et al.). It represents a case study using the lens of gender studies to investigate the role of early female laboratory neuroscientists at the beginning of the twentieth century.

It is interesting to note that the narrative of most of these articles reflects at least one scientific controversy, such as that of brain localization beginning in the Middle-Ages and Renaissance (Parent), which dominates the field of neuroanatomy in the early twentieth century and persists in today's questions about the nature of cortical function. Other sociological aspects are, respectively, studied by Desmoulin-Canselier and Moutaud, Lorch, and Stahnisch, in investigating debates about translational research, actor networks, and epistemological attitudes in public discourses about the role and status of science in early modern and in modern societies.

These historiographical perspectives generally lead the contributing authors of this special issue to elaborate on and correct traditional historical narratives which have sometimes been presented as myths introduced by dominant scientists to promote their own scientific successes. This is particularly visible in those articles concerned with the historical retelling of the development of the DBS technique, forgotten aspects of Loeb's early research in Europe, Bastian's disappearance into historical oblivion with the ascendancy to major prominence of John Hughlings Jackson (1835–1911) at the beginning of the twentieth century, the overlooked role of neurophysiologists in the defense of Darwinism and materialism, or Cajal's forgotten and generally positive attitude toward women scientists at the end of his career.

Most importantly, the contributions assembled in this special issue explicitly bridge past perspectives with areas of live current debate. They demonstrate how history can inform present understandings by exploring historical neuroscientific issues, placed in a theoretical frame, and contextualized with sociological and epistemological considerations. Several of these articles employ sociological concepts from actor network theory in their historical approaches to the description and analysis of neuroscience as an interdisciplinary scientific endeavor with a high degree of institutionalization, be it at the level of research laboratories, institutes, international organizations. Further epistemological perspectives draw on the complex interdisciplinary relations and the contingency of their respective place and time. For example, Eling and Finger link Gall's philosophical understanding of brain localization in the late 19th century to the theory and practice of present-day brain imaging studies. Lorch demonstrates how Bastian conceived of theoretical localization models for their utility to understand the relationship of clinical patient observations and with bedside nosography. At the same time, Bastian proposed that the neurophysiological architecture was in the form of a network of connected regions with individual variation, thereby bringing together theory-laden observations and hypotheses. Such issues are topics of live debate in current epistemological conceptualizations, as far as present epigenetic neurocognitive models are concerned.

Overall, this *Frontiers in Neuroanatomy* special issue emphasizes the valuable contribution made by the scholarship in the history of the neurosciences in its wide-reaching analyses. It offers us new perspectives, assists in better understanding neuroscientific concepts and practices today, and provides rich and detailed exemplars of how consideration and engagement with past historical contexts can inform current theoretical and practical debates in neuroscience.

## Author Contributions

J-GB, ML, and FS contributed equally to writing this Editorial of the Research Topic on History of Neuroscience that they edited from 2018 to 2019. All authors contributed to the article and approved the submitted version.

## Conflict of Interest

The authors declare that the research was conducted in the absence of any commercial or financial relationships that could be construed as a potential conflict of interest.
